# Supplementary figures and images for: Influence of Messa di Voce speed on vocal stability of professionally trained singers
Source: PLoS One. 2025 Jun 10;20(6):e0325284. doi: 10.1371/journal.pone.0325284 (PMC12151380; doi:10.1371/journal.pone.0325284)

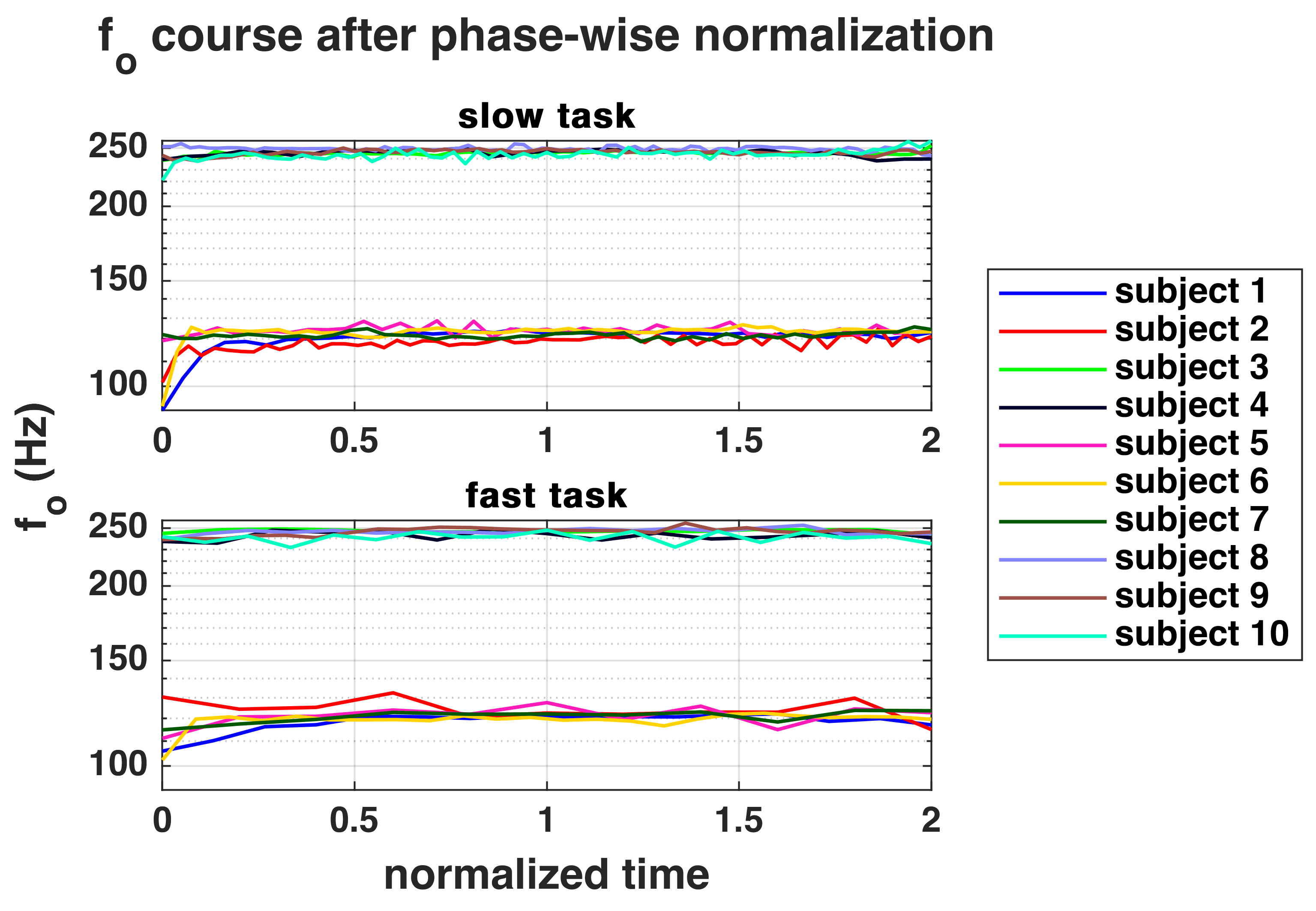

Supplement: S1 Fig — The SPL apex of the MdV is represented by time point 1, the end of the task is represented by 2. Note that 0–1 and 1–2 do not necessarily represent similar durations. (TIF) [file pone.0325284.s001.tif]
