# Supplementary material for: Influence of Messa di Voce speed on vocal stability of professionally trained singers
Source: PLoS One. 2025 Jun 10;20(6):e0325284. doi: 10.1371/journal.pone.0325284 (PMC12151380; doi:10.1371/journal.pone.0325284)
Supplement: Attachment — Submitted filename: Response to Reviewers.docx [file pone.0325284.s003.docx]

Dear Editor and Reviewers,

Thank you for your positive and helpful assessment. Below, we address every issue raised.

Kind regards,

Marie Köberlein and colleagues

1. Please ensure that your manuscript meets PLOS ONE's style requirements, including those for file naming. The PLOS ONE style templates can be found at https://journals.plos.org/plosone/s/file?id=wjVg/PLOSOne_formatting_sample_main_body.pdf and https://journals.plos.org/plosone/s/file?id=ba62/PLOSOne_formatting_sample_title_authors_affiliations.pdf

Answer: We have adjusted the manuscript to PLOS ONE’s style requirements.

 2. We noticed you have some minor occurrence of overlapping text with the following previous publication(s), which needs to be addressed:

https://pubmed.ncbi.nlm.nih.gov/39883711/

In your revision ensure you cite all your sources (including your own works), and quote or rephrase any duplicated text outside the methods section. Further consideration is dependent on these concerns being addressed.

Answer: Since the mentioned publication is the previous publication of our same author team, regarding the same topic and thus being closely linked to the current manuscript, some identical phrases occurred. We checked the document and rephrased these sentences.

 3. Please review your reference list to ensure that it is complete and correct. If you have cited papers that have been retracted, please include the rationale for doing so in the manuscript text, or remove these references and replace them with relevant current references. Any changes to the reference list should be mentioned in the rebuttal letter that accompanies your revised manuscript. If you need to cite a retracted article, indicate the article’s retracted status in the References list and also include a citation and full reference for the retraction notice.

Answer: The reference list has been checked.

Reviewer's Responses to Questions

**Comments to the Author**

1. Is the manuscript technically sound, and do the data support the conclusions?

The manuscript must describe a technically sound piece of scientific research with data that supports the conclusions. Experiments must have been conducted rigorously, with appropriate controls, replication, and sample sizes. The conclusions must be drawn appropriately based on the data presented.

Reviewer #1: Partly

Reviewer #2: Yes

2. Has the statistical analysis been performed appropriately and rigorously?

Reviewer #1: I Don't Know

Reviewer #2: I Don't Know

3. Have the authors made all data underlying the findings in their manuscript fully available?

The [PLOS Data policy](http://track.editorialmanager.com/CL0/http:%2F%2Fwww.plosone.org%2Fstatic%2Fpolicies.action%23sharing/1/010f0196451dec5b-9923cf83-eb02-44a7-a05f-e409e44da64d-000000/TsCKErD6dOK8SP_hIo75ZhT5Zd-uHwGpmx2ZRKEwJtE=207) requires authors to make all data underlying the findings described in their manuscript fully available without restriction, with rare exception (please refer to the Data Availability Statement in the manuscript PDF file). The data should be provided as part of the manuscript or its supporting information, or deposited to a public repository. For example, in addition to summary statistics, the data points behind means, medians and variance measures should be available. If there are restrictions on publicly sharing data—e.g. participant privacy or use of data from a third party—those must be specified.

Reviewer #1: No

Answer: Due to the ethical votum, we cannot provide the original recordings. However, the data points are available in the data repository: <https://doi.org/10.5281/zenodo.14900471>

Reviewer #2: Yes

4. Is the manuscript presented in an intelligible fashion and written in standard English?

PLOS ONE does not copyedit accepted manuscripts, so the language in submitted articles must be clear, correct, and unambiguous. Any typographical or grammatical errors should be corrected at revision, so please note any specific errors here.

Reviewer #1: Yes

Reviewer #2: Yes

5. Review Comments to the Author

Reviewer#1

Areas for Improvement

As the task requires singers to maintain a stable pitch, rhythm, and intensity. However, the study does not include contour plots for fundamental frequency, rhythm, and intensity, which are crucial to verifying the stability of these parameters. Instead, the authors only plot sound pressure level (SPL) over time for the slow and fast tasks. The authors should plot these parameters to confirm that they remained stable throughout the task, as this is a key assumption in their analysis.

Answer: We added plots of fundamental frequency to the Supporting material (S1 Fig). Regarding rhythm, we think that a MdV does not really have a rhythm, since it is only one sustained tone/note/pitch. The plot of SPL over duration can show a certain “rhythm”, i.e., the distribution of crescendo and decrescendo. We disagree that the task requires maintaining a stable intensity. The principle of MdV is to vary intensity, i.e., SPL, over time. The most objective way to depict intensity is the SPL.

The introduction mentions MFDR as an important indicator, yet the study relies solely on EGG-based measurements.
Suggestion: It would be beneficial to extract voice source parameters such as MFDR, closing quotient, and opening quotient from the singing voice itself rather than exclusively from EGG. This would provide a more comprehensive analysis of how voice source parameters impact Messa di Voce (MdV).

Answer: The study does not solely rely on EGG measurements, recall Table 2. Especially the closing quotient which is strongly linked to Maximum Area Declination Rate and also MFDR, but also the open quotient, are derived from High-speed-video material, delivering a precise Glottal Area Waveform (GAW).

Regarding your suggestion: With exclusion of the OQ, we do not believe that the extraction of Closing Quotient or MFDR out of the EGG signal would be meaningful. Instead, especially MFDR would have to be calculated by means of construction of the flow glottogram (thus from the audio signal). However, this is under strong discussion because of the assumption of a linear source/filter relation. Since there could be non-linearities occurring during the experiment (this was the reason to calculate the sample entropy as non-linear measure), we would doubt such results. In the presented data set, there is an inclusion of the GAW derived Closing Quotient which is related to both SPL and MADR.

Quality of Figures: The figures, especially the last few, are of poor quality, making them difficult to read. The authors should improve the resolution and clarity of the figures to enhance readability and ensure that critical details are not lost.

Answer: The editor confirmed that the bad resolution was due to the compression applied by the PLOS ONE management system and that the quality of the original files is decent. We have now increased the font sizes for better readability.

Integration of Figures with Text: There is a lack of coherence between the figures and the text. Some figures are not well-referenced in the discussion, making it difficult to follow the analysis. The authors should directly reference figures within the text and provide a clearer explanation of their relevance to the discussion.

Answer: We tried to improve the issue by referencing the figures in the discussion.

Scientific Backing for Findings:
The authors should provide stronger scientific explanations for why they obtained their results. For example, why does sample entropy indicate higher laryngeal stability? Why is vibrato not correlated with SPL in this cohort?

Answer: The Sample Entropy is a measure for the complexity of physiological time-series signals. A higher SE indicates lesser laryngeal stability and higher irregularity. We added a comment in the discussion and literature on this [37, 38].
As we state at the end of the discussion, it remains unclear why the cohort mostly avoided vibrato and why there was no or even a negative correlation in some cases. It might be due to the experimental circumstance where subjects tried to sing a clearly defined *f*_o_ on exact length in order to only change one variable (SPL).

Suggestion: A more thorough discussion of underlying physiological mechanisms and previous literature would strengthen the scientific validity of the conclusions.

Answer: We added once again the control mechanisms for SPL control and some literature [48-51].

The scatter plots presented in the later sections should be examined more critically. Some outliers and spurious values are present in SPL, RAP, sample entropy, and other measures. The authors should analyze whether these values result from individual variations among singers or measurement inconsistencies. Investigating potential correlations among different parameters could also provide deeper insights into Messa di Voce (MdV) mechanisms.

Answer: We absolutely agree. To be honest, looking at the individual courses and measurements we do not find any good explanation for some outliers. It should be noted, however, that also these outliers are really in the very low physiological range and therefore expectable. We added “It is noticeable that there were – in contrast to OQ_GAW_, OQ_EGG_ and ClQ – some individual differences and outliers. However, as can be seen in Fig. 5, even such outliers did not exceed the value of .12 by a great amount and are therefore in the lower physiological range.”

This study explores an interesting and relevant topic in voice science, providing valuable insights into Messa di Voce (MdV) and voice stability in professional singers. However, several areas require improvement before publication. The lack of fundamental contour plots, limited use of MFDR from singing voices, poor figure quality, and insufficient scientific backing for findings weaken the study’s overall impact. Addressing these concerns and integrating the suggested improvements would enhance the clarity, coherence, and robustness of the study.

Answer: Thank you for your assessment. We have addressed the points raised.

Reviewer #2: 

ABSTRACT
The abstract is well structured and provides relevant information that is consistent with the research objective.
However, the way the research objective is described is vague. For example, I cannot understand whether what the authors want to investigate is the “effect” of task speed on some other variable. The authors' writing of the objective does not make it clear that this association is being investigated. Therefore, I suggest that they review the way they present the objective. The aim seems to be much clearer in the title of the work and could be transposed (with the necessary adjustments in the writing) to the abstract and to the end of the introduction (where the objective is described).

Answer: Thank you, we tried to improve the issue as suggested.

INTRODUCTION
The authors provided an adequate contextualization of the topic. The most relevant studies and research hypotheses were presented.
However, what problem would be solved if this hypothesis were confirmed is unclear. In this sense, the authors should present in the introduction the context or problem that will be solved with this knowledge. Although the study's rationale is well presented, its relevance is unclear to the reader.

Answer: You are right. It is of basic interest for voice training if the speed of a certain exercise has effects on stability parameters and consequently how the exercise should be executed ideally. We added: “The outcomes could provide information for different fields of voice training on which speed of the MdV task should be applied for different purposes and how it could be mastered.”

METHOD

The authors did not define the study design. It is not clear how the singers were recruited nor what the exclusion criteria would be for participants.

Answer: We added information on this:
“This prospective observational cohort-study follows the STROBE guidelines.”
“The participants were recruited from the local university of music community through personal contact. The prerequisite was at least a bachelor's degree and vocal health, which was verified by endoscopy and Dysphonia Severity Index (DSI) assessment.” We also added the subjects’ DSI values to Table 1.

The authors need to provide more information to justify some choices. For example, please explain why the vowel [i] was chosen and not another vowel. It may seem obvious, but it is not so for all potential readers of this manuscript.

Answer: We added: “The vowel [i] was chosen, since the associated tongue position with tongue elevation and forward shift provides the best visibility on the vocal folds.”

A detailed description of the data collection procedures is missing, which would allow the study to be reproducible. For example, how was the duration of the task controlled? Was it self-monitored? What is the justification for choosing phonation at these frequencies?

Answer: We added:
“The duration of the task was not controlled but self-monitored by the subjects. This limitation was accepted in the study design because an acoustic metronome would have disturbed the audio recordings, and the application of a visual metronome was impossible due to the examiner standing right in front of the participants and the space-consuming technical equipment around them.”

“The *f_o_* were chosen in the region of the speaking voice, since here the vocal apparatus works most efficiently and the probability of other disruptive factors’ influence, e.g. higher tension for higher pitches, would be minimized.”

Was the HSV, voice, and EGG data collected simultaneously or sequentially?

Answer: As stated in the Material and methods section, everything was recorded simultaneously to provide comparability of the parameters. We exchanged the word “parallel” by “simultaneously” to make it clearer.

What justifies the use of Spearman's correlation? Were the data non-normally distributed? Was the sample size small? While they used Spearman's correlation (a non-parametric test), the authors used ANOVA (a parametric test). I would like to hear the authors' justifications for this.

Answer: We used Spearman correlation to assess associations without relying on the normality of the raw data. We then used ANOVA to examine differences between group means. Even if the raw data are not perfectly normally distributed, the central limit theorem and the robustness of ANOVA mean that moderate deviations from normality may not be problematic when looking at group differences.

There are also non-parametric tests similar to ANOVA, but those can either not handle multiple main factors (like age, sex, task type), require a very high number of samples or would rely on a complete statistical modeling process which would even put more requirements.

RESULTS
The results were presented in an appropriate and didactic manner. The authors reviewed the hypotheses and discussed the results found.
I recommend that the authors comment on the practical implications of the findings of this study for vocal training, whether through the intervention of singing teachers or SLPs.

Answer: Thank you for the positive and helpful assessment. We added another subsection:

“Practical implications

The observations that the untrained singers showed correlations of SPL and RAP, and struggled before the apex of the fast task, while the professionals did not, leads to the assumption that as prerequisite for a MdV, one must master stable phonation on one pitch at the different levels of SPL with a good vocal fold closure. These can afterwards be linked smoothly in slow MdV tasks which provide more time for coordination. While slow MdV poses a challenge on breath control, fast MdV needs faster laryngeal coordination. Thus, for advanced training, different speeds should be applied with regard to the training’s focus.”
